# Triangular fibrocartilage complex injury in cardiopulmonary resuscitation providers: A case report

**DOI:** 10.1097/MD.0000000000044532

**Published:** 2025-09-26

**Authors:** Yijun He, Cheng Luo, Jiongfeng Huang

**Affiliations:** a Department of Osteoarthropathy and Sports Medicine, The Affiliated Panyu Central Hospital, Guangzhou Medical University, Guangzhou, Guangdong, PR China.

**Keywords:** cardiopulmonary resuscitation, case report, DRUJ injury, TFCC injury, work-related musculoskeletal injuries

## Abstract

**Rationale:**

Cardiopulmonary resuscitation (CPR) is physically demanding and may result in work-related musculoskeletal injuries in healthcare providers. We report a case of triangular fibrocartilage complex (TFCC) and dorsal distal radioulnar ligament injuries sustained by an orthopedic resident during prehospital CPR events, to increase awareness of this underrecognized occupational hazard.

**Patient concerns:**

A 31-year-old orthopedic resident performed prolonged CPR twice in one shift and subsequently developed marked tenderness, instability, and pain in the left wrist with pronation, as well as a sensation of clicking subluxation. A second physician recalled persistent wrist pain after CPR in previous years.

**Diagnoses:**

Imaging demonstrated a tear of the TFCC and dorsal distal radioulnar ligament as well as joint effusion and extensor carpi ulnaris sheath edema in the affected wrist. The retrospective magnetic resonance imaging of the second physician indicated chronic TFCC change.

**Interventions:**

Conservative management with nonsteroidal anti-inflammatory drugs, plaster splint immobilization, and subsequent wrist bracing was implemented for the resident; self-treatment with nonsteroidal anti-inflammatory drugs and wrist brace was reported by the second physician.

**Outcomes:**

The orthopedic resident achieved resolution of instability after 6 weeks with restoration of full wrist motion and strength by 2 years. The second physician’s symptoms persisted for 2 years before mostly resolving.

**Lessons:**

Musculoskeletal injuries, including TFCC and distal radioulnar ligament tears, may occur in CPR providers but are often underrecognized and underreported. Early recognition, appropriate management, and preventive strategies such as alternating roles, prompt initiation of automated compression devices, and ergonomic device placement may mitigate injury risks. Encouraging timely reporting and self-care among healthcare professionals is essential for provider well-being and care quality.

## 1. Introduction

Cardiopulmonary resuscitation (CPR) is a life-saving technique employed in emergency situations when an individual’s heartbeat or breathing has ceased.^[1]^ Although the procedure can be immensely beneficial for the patient, it is not without risks for the person performing it. According to the literature, CPR can impose significant work-related musculoskeletal (MSK) injuries on the performer, affecting different body parts such as the back, knee, shoulder, and wrist.^[2–7]^ These injuries vary from strains, and sprains to tendon tears, which might impose lasting consequences on the performer’s overall health and ability to work.

Regrettably, occupational MSK injuries are often less concerning and diagnosed late due to various factors. Some studies have shown that medical practitioners tend to treat themselves and may not seek immediate medical attention.^[8–10]^ This can lead to a delay in appropriate treatment and may exacerbate the injury. Additionally, the high physical demands and time-sensitive nature of emergencies may contribute to the under-reporting and under-treatment of these injuries, further increasing the risk of long-term complications.

CPR performers, in prehospital situations particularly, are at risk for a variety of MSK injuries. These risks can be attributed to factors such as repetitive forceful compressions, awkward body positioning, and insufficient breaks between resuscitation attempts. This case report describes a wrist injury sustained during CPR, emphasizing the importance of early recognition and appropriate management of MSK injuries arising from this physically demanding procedure.

## 2. Case 1

**Figure 1.**
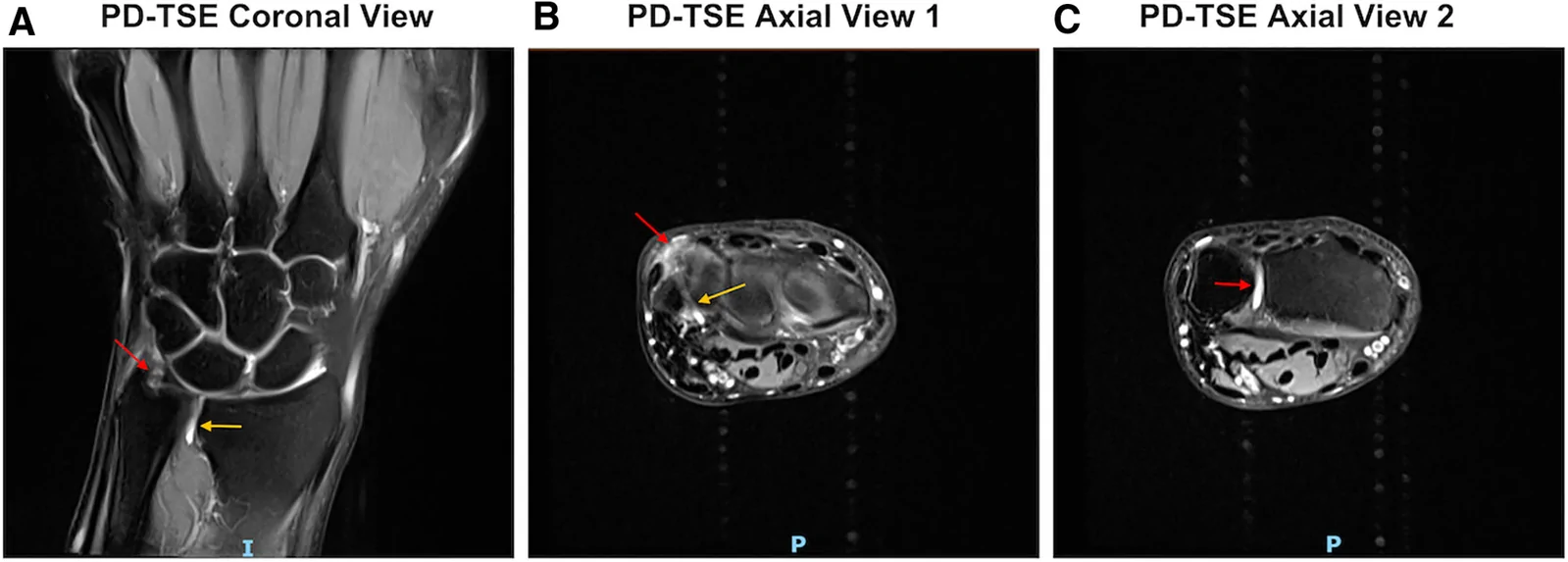
Magnetic resonance imaging of case 1: (A) coronal view demonstrating a long liner signal extending from the upper portion of the TFCC to the bottom, indicating an avulsion TFCC injury (red arrow). Besides, joint effusion was present within the DRUJ (orange arrow); (B) Axial view demonstrating long signals from dorsal to volar side ulnarly (red and orange arrow), consistent with the tear in the coronal view; (C) Joint effusion in DRUJ extending to the dorsal side (red arrow), indicating possible tear of dorsal ligament of the DRUJ. DRUJ = distal radioulnar joint, TFCC = triangular fibrocartilage complex.

A 31-year-old orthopedic resident trained with advanced cardiac life support encountered 2 separate cardiopulmonary resuscitations (CPR) events during a 12-hour Emergency Department shift in the prehospital setting. The resident performed CPR for a minimum of 30 minutes for each individual, with an approximate 6-hour interval between the 2 sessions. Following the initial one, the resident experienced mild tenderness in the left wrist but did not express significant concern. Eight hours after completing the second CPR session, the resident perceived marked tenderness over the dorsal and dorsoulnar aspects of the left wrist, accompanied by a sensation of instability and pain in pronation. The resident recalled the feeling of a mild clicking subluxation during antebrachial pronation, leading to the suspicion of a distal radioulnar joint subluxation. The resident had a nonspecific prior medical, family, or psychosocial history related to the injury or any previous history of musculoskeletal injuries including fractures or sprains. Physical examination revealed noticeable wrist joint swelling and pain provoked by forced wrist extension and pronation. A snapping sensation over the DRUJ was present during passive forearm supination and pronation and a positive extensor carpi ulnaris test was revealed. However, neither a positive fovea sign nor a piano key sign was observed. An immediate 3.0T magnetic resonance imaging examination disclosed joint effusion within the distal radioulnar joint (DRUJ) and the ulnocarpal joint. Concurrently, a linear long signal intensity was observed transecting the triangular fibrocartilage complex (TFCC) disc proper, along with a similar long signal intersecting the dorsal radioulnar ligament in the axial plane (Fig. 1A and B). These findings are consistent with a tear of the TFCC and the dorsal distal radioulnar ligament. Besides, an edematous signal was also present within the sheath of the extensor carpi ulnaris (Fig. 1C). In the coronal plane, a high signal intensity was detected on the ulnar aspect of the TFCC, suggesting an acute avulsion of the TFCC from the ulnar styloid (Fig. 1A). A conservative treatment approach was attempted as requested by the resident, which included oral administration of nonsteroidal anti-inflammatory drugs (NSAIDs) and plaster splint immobilization. The affected limb was immobilized in 90-degree elbow flexion and forearm supination, extending from the metacarpal region to the mid-brachial region, for 6 weeks, during which a sick leave was applied. Then, a rigid wrist brace was applied for the following 6 weeks. The conservative management was well-tolerated by the resident. No adverse and unanticipated events were encountered. During the follow-up evaluation, the resident reported a resolution of the previously experienced sense of instability after 6 weeks and restoration of full range-of-motion, grip and forearm rotation strength 2 years after the onset of injury (Table 1).

**Table 1:** Timeline demonstration of case 1.

Timepoint	Event/ procedure	Observations/ interventions
Start of 12-h ED shift	First CPR Session	30 min of manual CPR performed; Mild left wrist tenderness noted
6-h after first session	Second CPR Session	Another 30 min of manual CPR session performed
8-h post second session	Onset of marked symptoms	Marked tenderness over the dorsal/dorsoulnar wrist; Sensation of instability and clicking during forearm movements
Immediately after marked symptom onset	Diagnostic imaging	3.0T MRI conducted; Findings consistent with TFCC tear and dorsal distal radioulnar ligament tear; Edema noted in extensor carpi ulnaris tunnel
Initiation of treatment	Conservative management	Oral NSAIDs, plaster splint immobilization (6 w k elbow at 90° flexion, forearm in supination)
6-w k posttreatment initiation	Reassessment and additional support	Application of a rigid wrist brace for the following 6 wk
6-wk post-immobilization	Follow-up evaluation	Resolution of the sense of instability
2-yr post-injury	Final outcome	Full range-of-motion and normal strength restored

CPR = cardiopulmonary resuscitation, MRI = magnetic resonance imaging, NSAIDs = nonsteroidal anti-inflammatory drugs, TFCC = triangular fibrocartilage complex.

## 3. Case 2

**Figure 2.**
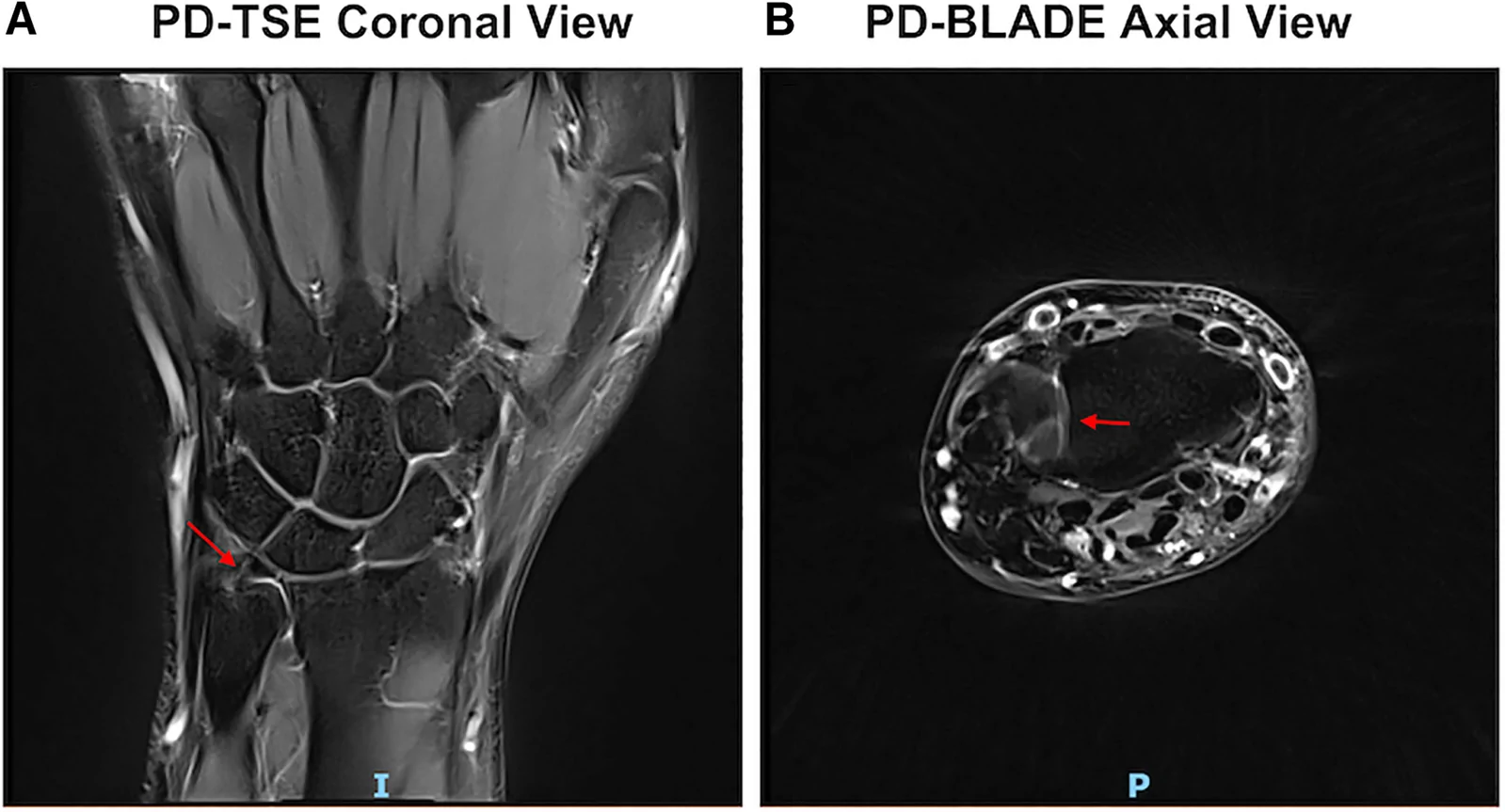
Magnetic resonance imaging of the case 2. (A) Coronal view demonstrating moderate-to-long signal intensity extending from the upper disc surface to the bottom, indicating a possible chronic TFCC injury (red arrow); (B) moderate-to-long signal runs diagonally through the disc (red arrow), consistent with the morphology in the coronal view. TFCC = triangular fibrocartilage complex.

A 32-year-old male physician, upon learning about the first case, recalled experiencing left wrist pain following 2 consecutive CPR sessions 3 years prior. The pain worsened with increased upper-limb manual activities and subsided intermittently with rest. After approximately 1 month of continuous pain, the symptoms persisted intermittently for 2 years before mostly resolving at the time of reporting. The physician self-managed his condition by taking nonsteroidal anti-inflammatory drugs (NSAIDs) and wearing a wrist brace for support and pain control during daily activities. Physical examination revealed mild tenderness on the ulnar side of the wrist, which was not exacerbated by ulnar deviation. Pain-free range-of-motion and strength was documented. A retrospective magnetic resonance imaging revealed a linear, long signal traversing the disc (Fig. 2A and B), possibly indicative of a previous triangular fibrocartilage complex (TFCC) tear. The physician did not report to supervisor and receive any formal treatment.

## 4. Discussion

The presented case highlights that CPR performers can sustain wrist injuries, which can impair their function, affect their quality of life and even work performance. Triangular fibrocartilage complex (TFCC) injuries and distal radioulnar joint (DRUJ) instability are related wrist conditions involving the structures that provide stability and support, particularly during forearm rotation and weight-bearing movements.^[11,12]^ The TFCC is a complex network of ligaments, cartilage, and a fibrocartilage disc connecting the distal ends of the radius and ulna, while the DRUJ is a joint formed by their articulation. Injuries to the TFCC can result from acute trauma or chronic degeneration, while DRUJ instability often occurs due to damage or weakening of the supporting ligaments, sometimes associated with TFCC injuries.^[13]^ Both conditions can cause pain, swelling, limited range-of-motion, and a sense of instability or “clicking” during forearm rotation, often requiring conservative treatment or surgical intervention for optimal recovery. To execute a conventional CPR chest compression, the rescuer positions 1 hand on top of the other, interlocking their fingers. Generally, both wrists are hyperextended, and both forearms are in a pronated position, supporting the rescuer’s body weight throughout each compression. This posture is akin to the one that results in a torn TFCC and DRUJ instability due to a fall on an outstretched hand. Previous evidence suggested high compressive forces applied to the wrist during chest compressions, which can exceed 600N.^[14]^ The stress placed on rescuers’ wrists during CPR chest compressions, even at <10% of the total force, can significantly damage the scapholunate ligament in the wrist.^[2]^ In another study, all participants reported feeling pain or discomfort in at least one joint within 5 minutes of performing CPR chest compressions. The wrist joint was the most mentioned area of pain, with every participant reporting wrist discomfort.^[15]^ Therefore, the possible mechanisms of wrist injuries in CPR performers might be attributed to the high compressive forces applied to the wrist during each chest compression, repetitive loading, and unloading cycle. Performing CPR can be physically demanding. Unlike athletic activities, where appropriate warm-up exercises can be conducted, CPR necessitates an immediate response. Consequently, sudden, prolonged, or repetitive compressions may place strain on muscles, ligaments, and joints.

Nonetheless, it is noteworthy that the individual in this instance sustained an injury on this occasion, despite having been participating in yearly training and having numerous CPR experiences throughout their career. Guidelines suggest that rescuers may experience a decline in their ability to deliver chest compressions at an adequate depth after approximately 2 minutes of performing CPR due to fatigue.^[1,16,17]^ Furthermore, muscle fatigue significantly contributes to the development of sports injuries through muscle and postural compensation. Fatigued muscles have a reduced capacity to generate force and maintain stability, leading to athletes inadvertently adopting compensatory movement patterns. These compensatory actions often involve overloading adjacent muscles or modifying joint mechanics to preserve functionality. Such biomechanical alterations place increased stress on muscles, ligaments, and joints, ultimately heightening the risk of injury.^[18,19]^ In the current case, the resident performed 2 CPR sessions within a single shift, which took place in prehospital settings where awkward positioning was unavoidable. This suggests that fatigue may have played a role in the incident. Notably, previous cases have reported a rotator cuff tear after performing 3 compressions in 1 shift, and a long head of biceps tendon tear after a pediatrician performed 45 minutes of continuous compression.^[6,20]^ Consequently, it is essential to remember that rescuers should alternate roles every 2 minutes, or even sooner, if possible, to prevent such unintended injuries.

It is important to consider that physicians, due to the demands of their profession, might not report or seek medical attention promptly, potentially resulting in unfavorable outcomes.^[6,8–10]^ In this case, the orthopedic training background allowed the resident to accurately identify the underlying pathology and pursue appropriate imaging and treatment. However, this may not be the case for physicians and surgeons who lack familiarity with such conditions. After case 1 was reported to the department supervisor, another emergency room physician also disclosed having experienced wrist pain following CPR that persisted for 2 years, but self-treated with NSAIDs and a brace without undergoing imaging studies. The unique challenges faced by physicians in their demanding profession can sometimes lead to delayed or under-reporting and treatment of their health issues, increasing the risk of adverse outcomes. While the orthopedic training background of the resident, in this case, facilitated a timely diagnosis and appropriate intervention, it is crucial to recognize that not all may have the same level of familiarity with such conditions. The disclosure of a similar case by another emergency room physician highlights the importance of encouraging healthcare professionals to prioritize their own well-being and seek timely medical care when necessary, ensuring both their health and the continued provision of high-quality care for their patients.

Furthermore, there are several innovative strategies have been proposed to mitigate the risk of injuries among healthcare providers while maintaining the effectiveness of CPR. One such strategy involves alternating roles during CPR. By sharing the workload and rotating roles, providers can reduce the risk of injury due to overexertion, maintain optimal compression quality, and minimize the risk of musculoskeletal injuries. Besides, the prompt initiation of automated compression devices can play a significant role in reducing provider injuries. These devices consistently deliver high-quality chest compressions without causing fatigue or injury to healthcare providers. By improving patient outcomes and protecting providers from potential injuries associated with manual compressions, automated compression devices can enhance safety during CPR.^[21]^ Moreover, the ergonomic placement of resuscitation devices is crucial for reducing the risk of injury among healthcare providers.^[22]^ Proper positioning and alignment of equipment minimize awkward postures and movements that may contribute to musculoskeletal injuries.

The case report under discussion presents an intriguing first-time documentation of a wrist injury involving the TFCC and DRUJ following CPR. The identification of a previously unreported injury pattern is a valuable contribution to the medical literature, as it may prompt further investigation and increased awareness among healthcare professionals. However, the findings in this single case reports are inherently lack in the generalizability. While the described injury may represent a genuine risk associated with performing CPR, it is difficult to determine the prevalence or significance of this specific injury pattern based on a single case. It is possible that the injury could have been coincidental or related to other factors not directly associated with CPR.

## 5. Conclusion

In conclusion, this case report highlights the potential risks of musculoskeletal injuries, particularly wrist injuries, associated with performing CPR for healthcare providers. It emphasizes the importance of early recognition, timely reporting, and appropriate management of these injuries to prevent long-term complications. Furthermore, the implementation of innovative strategies, such as alternating roles during CPR, prompt initiation of automated compression devices, and ergonomic placement of resuscitation equipment can help to mitigate the risk of injuries among healthcare providers. Encouraging healthcare professionals to prioritize their own well-being and seek timely medical care is essential for their health and the continued provision of high-quality care for their patients. By raising awareness about the potential risks and promoting safer CPR practices, we can work towards creating a safer environment for both healthcare providers and patients.

## Author contributions

**Conceptualization:** Cheng Luo, Jiongfeng Huang.

**Funding acquisition:** Yijun He.

**Investigation:** Yijun He, Cheng Luo.

**Methodology:** Jiongfeng Huang.

**Supervision:** Cheng Luo, Jiongfeng Huang.

**Writing – original draft:** Yijun He.

**Writing – review & editing:** Yijun He.

## References

[1] Travers AH Perkins GD Berg RA; Basic Life Support Chapter Collaborators. Part 3: adult basic life support and automated external defibrillation. Circulation. 2015;132:S51–83. 10.1161/CIR.0000000000000272 26472859

[2] Curran R Sorr S Aquino E. Potential wrist ligament injury in rescuers performing cardiopulmonary resuscitation. J Emerg Trauma Shock. 2013;6 :123–5. 10.4103/0974-2700.110776 PMC3665060 23723622

[3] Dainty RS Gregory DE. Investigation of low back and shoulder demand during cardiopulmonary resuscitation. Appl Ergon. 2017;58:535–42. 10.1016/j.apergo.2016.04.013 27179543

[4] Jones AYM. Can cardiopulmonary resuscitation injure the back? Resuscitation. 2004;61:63–7. 10.1016/j.resuscitation.2003.12.007 15081183

[5] Jones AYM Lee RYW. Cardiopulmonary resuscitation and back injury in ambulance officers. Int Arch Occup Environ Health. 2005;78:332–6. 10.1007/s00420-004-0577-3 15827758

[6] Jackson CE Sturrock R. Resuscitation shoulder – a complication of cardiac arrest. Rheumatology (Oxford). 2007;46:1040–1. 10.1093/rheumatology/kem047 17409130

[7] Friedenberg R Kalichman L Ezra D Wacht O Alperovitch-Najenson D. Work-related musculoskeletal disorders and injuries among emergency medical technicians and paramedics: a comprehensive narrative review. Arch Environ Occup Health. 2022;77:9–17. 10.1080/19338244.2020.1832038 33073742

[8] Cheung W Gullick J Thanakrishnan G. Injuries occurring in hospital staff attending medical emergency team (MET) calls--a prospective, observational study. Resuscitation. 2009;80:1351–6. 10.1016/j.resuscitation.2009.09.010 19837501

[9] Davis WT Sathiyakumar V Jahangir AA Obremskey WT Sethi MK. Occupational injury among orthopaedic surgeons. J Bone Joint Surg Am. 2013;95:e107. 10.2106/JBJS.L.01427 23925752

[10] Tan K Kwek E. Musculoskeletal occupational injuries in orthopaedic surgeons and residents. Malays Orthop J. 2020;14:24–7. 10.5704/MOJ.2003.004 PMC7156165 32296478

[11] Jawed A Ansari MT Gupta V. TFCC injuries: how we treat? J Clin Orthop Trauma. 2020;11:570–9. 10.1016/j.jcot.2020.06.001 PMC7384326 32742122

[12] Qazi S Graham D Regal S Tang P Hammarstedt JE. Distal radioulnar joint instability and associated injuries: a literature review. J Hand Microsurg. 2021;13:123–31. 10.1055/s-0041-1730886 PMC8440053 34539128

[13] Kim B Nho J-H Jung KJ Yun K Kim YH Yoon H-K. Traumatic triangular fibrocartilage complex injuries and instability of the distal radioulnar joint. J Korean Orthop Assoc. 2017;52:112.

[14] Baubin M Kollmitzer J Pomaroli A. Force distribution across the heel of the hand during simulated manual chest compression. Resuscitation. 1997;35:259–63. 10.1016/s0300-9572(97)00040-3 10203407

[15] Trowbridge C Parekh JN Ricard MD Potts J Patrickson WC Cason CL. A randomized cross-over study of the quality of cardiopulmonary resuscitation among females performing 30:2 and hands-only cardiopulmonary resuscitation. BMC Nurs. 2009;8:6. 10.1186/1472-6955-8-6 PMC2715393 19583851

[16] McDonald CH Heggie J Jones CM Thorne CJ Hulme J. Rescuer fatigue under the 2010 ERC guidelines, and its effect on cardiopulmonary resuscitation (CPR) performance. Emerg Med J. 2013;30:623–7. 10.1136/emermed-2012-201610 22851670

[17] Foo N-P Chang J-H Lin H-J Guo H-R. Rescuer fatigue and cardiopulmonary resuscitation positions: a randomized controlled crossover trial. Resuscitation. 2010;81:579–84. 10.1016/j.resuscitation.2010.02.006 20223578

[18] Drew MK Finch CF. The relationship between training load and injury, illness and soreness: a systematic and literature review. Sports Med. 2016;46:861–83. 10.1007/s40279-015-0459-8 26822969

[19] Jones CM Griffiths PC Mellalieu SD. Training load and fatigue marker associations with injury and illness: a systematic review of longitudinal studies. Sports Med. 2017;47:943–74. 10.1007/s40279-016-0619-5 PMC5394138 27677917

[20] Bhattacharya D Patra P Pilania RK Jindal AK. Tear of long head of biceps following cardiopulmonary resuscitation: a rare complication. BMJ Case Rep. 2019;12:e229218. 10.1136/bcr-2019-229218 PMC6536248 31122961

[21] Jennings PA Harriss L Bernard S. An automated CPR device compared with standard chest compressions for out-of-hospital resuscitation. BMC Emerg Med. 2012;12:8. 10.1186/1471-227X-12-8 PMC3441844 22734854

[22] Harari Y Riemer R Jaffe E Wacht O Bitan Y. Paramedic equipment bags: how their position during out-of-hospital cardiopulmonary resuscitation (CPR) affect paramedic ergonomics and performance. Appl Ergon. 2020;82:102977. 10.1016/j.apergo.2019.102977 31670157

